# Supplementary material for: Controlling fertilization and cAMP signaling in sperm by optogenetics
Source: eLife. 2015 Jan 20;4:e05161. doi: 10.7554/eLife.05161 (PMC4298566; doi:10.7554/eLife.05161)
Supplement: Figure 1—source data 1. — Data are given as mean ± s.d.; n = number of experiments. DOI: http://dx.doi.org/10.7554/eLife.05161.004 [file elife05161s001.docx]

Figure 1– source data 1. The Prm1-bPAC mouse model shows no change in fertility parameters. Data are given as mean ± s.d.; n = number of experiments.

|  | **wild-type** | **Prm1-bPAC** |
| --- | --- | --- |
| ratio testis weight/bodyweight (mg/g) | 3.34 ± 0.3 (n = 10) | 2.98 ± 0.4 (n = 5) |
| ratio epididymis weight/bodyweight (mg/g) | 0.71 ± 0.1 (n = 10) | 0.76 ± 0.1 (n = 5) |
| sperm count (per ml) | 2.74 ± 1.5 x 10^7^ (n = 15) | 2.31 ± 0.8 x 10^7^ (n = 15) |
| litter size at birth | 5.1 ± 0.7 (n = 13) | 6.7 ± 2.2 (n = 22) |
